# Developmental Prosopagnosia: A Review

**DOI:** 10.1155/2003/520476

**Published:** 2003-01-01

**Authors:** Thomas Kress, Irene Daum

**Affiliations:** Institute of Cognitive NeuroscienceDepartment of Neuropsychology Ruhr-University of BochumGermany

## Abstract

This article reviews the published literature on developmental prosopagnosia, a condition in which the ability to recognize other persons by facial information alone has never been acquired. Due to the very low incidence of this syndrome, case reports are sparse. We review the available data and suggest assessment strategies for patients suffering from developmental prosopagnosia. It is suggested that developmental prosopagnosia is not a unitary condition but rather consists of different subforms that can be dissociated on the grounds of functional impairments. On the basis of the available evidence, hypotheses about the aetiology of developmental prosopagnosia as well as about the selectivity of deficits related to face recognition are discussed.

## 1. Introduction

Developmental prosopagnosia (DP) is a very rare condition in which patients are not able to recognize familiar people on the basis of face information alone. In contrast to acquired prosopagnosia, in which the face recognition deficit evolves as a consequence of head trauma or stroke, DP manifests itself without any overt cause. There are no traumatic incidents in the medical history of affected patients. A definition of DP proposed by Jones and Tranel [45] entails the following criteria: the face recognition deficit is lifelong, it manifests itself in early childhood and it cannot be attributed to acquired brain damage. Other authors, however, include cases with documented developmental brain disease (see for example [2]). The first description of developmental prosopagnosia stems from McConachie [53]. She described AB, a 12-year-old girl, who was referred to psychosocial services because of social and psychological problems and was found to be severely impaired in face recognition. AB's mother also reported not to be able to recognise familiar faces, which was, however, not formally tested. McConachie [53] concluded, "... no hard evidence of neurological lesion was obtained, but the possibility of familial transmission was indicated".

Patients suffering from prosopagnosia have great difficulties recognizing other people. Failure to recognize close friends and family members is reported frequently. Some prosopagnosic patients are unable to recognize their own image in a mirror. Patients are usually able to make use of non-facial cues when attempting to identify a person. Often speech or gait is used to recognize others, and patients benefit greatly from the context in which people are met. Semantic knowledge about people is preserved which suggests that prosopagnosia is not due to general memory problems. In most cases, object recognition is well preserved and even if deficits in object recognition occur, they are much less pronounced than face processing impairments [3]. In every day life, prosopagnosia has severe effects on social interaction. For example, prosopagnosic people often find themselves in embarrassing situations when they fail to recognize familiar people at chance meetings. They tend to have fewer friends than other people and often withdraw from others. The problem often leads to feelings of guilt, especially in patients who are unaware of an organic cause of their deficit [23,68].

## 2. Development of face recognition

Normal face recognition undergoes rapid changes throughout the first year of life. Newborns can distinguish the face of their own mother from other faces within the first days of life, at least under certain cir-

ISSN 0953-4180/03/$8.00 © 2003 – IOS Press. All rights reserved cumstances ( [59], for a review see [57]. During the first weeks of life, face recognition abilities are somewhat unstable, depending on the assessment technique or the behavioural state of the newborn [57]. At the age of two months, there is activation in the fusiform gyrus when infants look at faces, although many other brain areas are also activated by faces that do not light up in adult brain imaging studies on face recognition [69]. Effects of face inversion have been observed in infants at the age of 4 months [28]. Infants between 3 and 7 months show a stabilized performance which is less task dependent than in younger infants. They are able to identify the gender of faces [13]. Between the 4th and the 9th month of age, a left visual field advantage evolves (e.g. [21]).

Findings of very early face preference as well as a report of a face recognition deficit due to a lesion at day 1 of age [30] have been taken as evidence for an innate face recognition system. Other authors have emphasised the role of experience for the development of face recognition. Due to extensive training with faces throughout the whole of life, humans become experts in face recognition and brain structures that mediate this skill are the same as those that mediate expert recognition of other visual stimuli (see for example [22,32]). It has been suggested that a strong social interest ensures that most people develop expertise in face recognition and that this social interest is mediated by the amygdala [35]. This account seems to largely ignore the early performance with faces exhibited by infants who did not have the opportunity to accumulate a large amount of visual expertise.

Other recent models incorporate two processes that are thought to be necessary for the development of a functional face recognition system [19,44,56]. They argue for an early process that "biases the newborn to orient to faces" and therefore guarantees input to a second experience-based face recognition system that "begins to influence behaviour at 6 to 8 weeks" [19]. The early process is functional from birth and therefore has to be hard wired. As a likely candidate for the neural substrate of this early process, subcortical structures, possibly the superior colliculus, have been proposed. Rather than "social interest", "visual interest" in faces may induce the infant's commitment to faces by ensuring input into the specializing neural systems for face recognition located in the ventral temporal cortex.

According to two-process theories of development of face processing, developmental prosopagnosia without documented brain damage appears as a result of one of two possible causes: First, a dysfunctional or absent mechanism responsible for passing input to developing visual areas or, second, dysfunction or absence of the cortices normally specialising in face processing. On the basis of the available evidence, no decision between these two alternatives seems possible. However, the observation that people suffering from developmental prosopagnosia are able to establish visual expertise in domains other than face recognition provides strong arguments for the absence of an early face orienting system, given that face recognition makes use of the same neural mechanism that mediates expertise for all visual stimuli. McNeil and Warrington [54] describe a patient with acquired prosopagnosia who is able to develop visual expertise for distinguishing between animals (sheep) despite his severe face recognition problems. There are however arguments that could lead to the assumption that the ability to acquire visual expertise per se depends on visual experience within a critical time period [42] in the same way as the ability to acquire speech depends on being exposed to speech within a certain time window (see for example [43]).

The presence of DP cases implies that plasticity of the face recognition system is limited. This lack of plasticity has been ascribed to the relatively small amount of tissue in the inferotemporal cortex that seems to be concerned with acquisition of visual expertise. Reduced plasticity might be the trade-off for the remarkable ability to make very fine discriminations among visual objects [57].

## 3. Models of face recognition

Increasing understanding of the different stages involved in recognition of individuals by their faces has led to the development of functional models of face recognition. The most influential of these models was proposed by Bruce and Young [9] and will be covered briefly. According to Bruce and Young [9], face recognition is based on several sub-processes that operate independently. At first, view-centred descriptions are derived from the visual input. Several physical aspects of faces can be analysed on the basis of this information including discrimination of age and gender, expression analysis as well as analysis of facial speech and matching of simultaneously presented faces (directed visual processing). To recognise an individual by the face, view-centred descriptions have to be translated into a viewpoint-independent description of the face. This process, termed structural encoding, generates a representation which allows comparison with familiar faces stored in memory. The structurally encoded representation is transferred to face recognition units (FRU) which in turn activate person identity nodes (PIN) in case the face is familiar. PINs allow access to semantic information about the person, such as profession, source of familiarity etc. Name generation is accomplished in a further processing step and can be dissociated from generation of semantic knowledge about the person. The model has been revised several times and has been adopted for an interactive activation approach to face recognition [10]. Other models relate the processes involved in face recognition to different structures in the temporal neocortex and the amygdala [66].

## 4. Functional deficits underlying prosopagnosia

### *4.1. Domain specificity*

The hypothesis of a specialized face recognition module as proposed by Bruce and Young [9] has been supported by neuropsychological case studies [30,54,72], electrophysiological studies (e.g. [5]) and fMRI experiments (e.g. [47]). Recently, doubt has been expressed about whether the neural correlates of face recognition are really unique for faces, or whether they represent brain activity that is linked to functions which mediate visual expertise on a more general level [32,33,63]. For an insight into the matters of the ongoing debate see for example [11] and [62].

### *4.2. Configural processing hypothesis*

According to the "configural processing hypothesis", the underlying impairment in prosopagnosia is the inability to get "an overview of an item as a whole in a single glance" [51]. This inability does not only affect face recognition but also other visual abilities that require the extraction of more than a single feature to specify the object's identity. If configural processing is deficient, errors of "visual underspecification" occur, that is specification of an object is carried out, although insufficient information is extracted for correct identification. Configural processing also explains why face matching might be intact in patients with prosopagnosia. Rather than processing the whole face at once, several features are compared sequentially. This results in correct responding, while performance is laboriously and slow. Configural processing can be tested with procedures that require inferring an object identity from a broken shape or a degraded picture. Failure in these tasks is considered to be a necessary but not sufficient condition for prosopagnosia. A recent review of cases suffering from acquired prosopagnosia [31] did not include any prosopagnosic patient unimpaired on tasks that tap configural processing, while one of the patients featuring in the present review (BC [23]) has been found to show normal configural processing. This can be seen as evidence against the configural processing hypothesis at least in cases of developmental prosopagnosia.

### *4.3. Level of categorization / expertise hypotheses*

The idea of the domain specificity of face recognition has been a matter of controversy. Some authors claim that face recognition requires a more complex level of categorization than object recognition, and prosopagnosia may reflect an impairment of within-class discrimination [34].

Second, humans are experts in recognising other humans on the basis of facial information. Prosopagnosia might result from a deficit in acquiring skills in visual information processing. People suffering from prosopagnosia would thus be expected to have difficulties in becoming experts for all classes of visual stimuli that require subtle discrimination [32].

The empirical evidence available so far does not allow a rejection of any of the above hypotheses, and the matter of the domain-specificity of face recognition is as yet unresolved.

## 5. Neural correlates of face recognition

The critical lesion location for prosopagnosia is still under debate. Acquired prosopagnosia is most frequently observed after bilateral damage. According to a summary by Farah [29] of 71 cases, 65% have a bilateral lesion, while 29% have a right sided lesion and only 6% have a left sided lesion. Farah [29] notes that due to limited resolution of brain imaging techniques, lesion extent is probably underestimated, and even more cases may have bilateral lesions. Damasio, Damasio and Van Hoesen [14] analysed autopsy data from 11 patients suffering from prosopagnosia and found lesions within fusiform and lingual gyri, often affecting the calcarine fissure with striate cortex and the hippocampal formation. These results are consistent with findings from functional brain imaging, which yielded activation for face processing tasks in the fusiform and inferior occipital cortices (see e.g. [36]). With respect to developmental prosopagnosia, there is only a single finding of abnormal brain structures [6], and it is unlikely that lesions in brain structures other than those listed above are responsible for the deficits observed in DP.

While acquired prosopagnosia is attributed to structural damage, there is little evidence for a structural brain deficit in DP (but see [6]). Most studies that provide brain scans do not report structural abnormalities (AB, EP, TA, VA). On the other hand, a number of studies have found functional deficits in DP both on the basis of EEG measures [6] and functional magnetic resonance imaging [36].

In normal subjects, the amplitude of a negative deflection peaking 170 ms following stimulus onset is enhanced for faces compared to other complex visual objects [5]. This finding is regarded as a marker of activity in neural networks responsible for face recognition. The so-called N170 component is insensitive to face familiarity [26] and selective attention [12], and enhanced and of longer latency in inverted compared to upright pictures of faces [25,64]. Bentin et al. [6] reported a reduced N170 difference between faces and houses in patient YT suffering from DP compared to a group of healthy control subjects; comparable results were observed for 2 DP cases by Kress and Daum [49]. In a patient with acquired prosopagnosia a reduced N170 for faces but not for houses was found [27]. Therefore, a smaller difference between N170 amplitude between faces and houses might serve as a marker of face recognition impairments.

Studies using functional brain imaging techniques (fMRI and PET) have identified a neural system for face recognition consisting of three regions in occipitotemporal visual extrastriate cortex [39]. A face specific region located in the inferior occipital cortex (OFA [37,40]) has been suggested to provide input to the face-specific areas located downstream. Representation of face identity has been ascribed to a face-specific area in the posterior fusiform gyrus [47,52]. A third region in the superior temporal sulcus has been associated with processing of variable aspects of faces such as direction of eye gaze [41]. Hadjikhani and de Gelder [36] reported that – unlike normal subjects – a patient suffering from DP did not activate regions in the fusiform or inferior occipital gyrus when looking at faces compared to objects. In patient YT, activation within the FFA was reported to be normal [38]. Conversely, Rossion et al. [61] reported activation within the region of the right FFA that was deprived of any input from the OFA, due to a lesion that affected the occipital face area. These results argue against a single feed forward mechanism that projects face-specific information sequentially from occipital to temporal cortices. Rather Rossion et al. [61] suggested that feedback connections from the right FFA to the right OFA may be necessary for normal face recognition.

## 6. Why study Developmental Prosopagnosia?

Developmental Prosopagnosia is a remarkable disorder because it appears in the absence of any overt brain lesion. It has been suggested, that a genetic factor rather than an undiscovered acquired brain lesion is responsible for the condition [16]. DP thus offers the opportunity to study face recognition deficits that are present from birth where face recognition abilities have never evolved.

In recent years, the number of reported cases of developmental prosopagnosia has increased. While there are at least 9 well documented cases published over a period of 26 years, there are considerable differences among these studies with respect to research techniques and the nature of assessed functions. Because DP is so extraordinarily rare, single case studies appear to be the only strategy to gain further insight into this condition. Moreover, evidence from different studies is often contradictory and inconsistent. Suggestions for future assessment of people suffering from DP would be useful to arrive at a better comparability between single case reports.

This review has two objectives. First, we aim to pool the empirical evidence on this very rare syndrome. Second, we aim to evaluate the testing procedures employed so far and to suggest procedures for future assessment.

## 7. Case Reports

Case descriptions play an important role in acquiring knowledge on rare neurological conditions such as DP. To our knowledge, there are 9 well-documented published cases for which a face recognition deficit was observed in the absence of a traumatic brain lesion (see Table 1 for a summary of these cases).

All cases discussed in this report fulfilled the criteria set by Jones and Tranel [45]. Neurological and neuropsychological testing of cases differed greatly between studies, which limits their comparability. Results are presented separately for functional domains, and suggestions for future assessment strategies will be made.

Table 1
Summary of patients with developmental prosopagnosia

| | | AB | BC | Dr. S | EP | HD | LG | TA | VA | YT |
|---|---|---|---|---|---|---|---|---|---|---|
| Reference | | McConachie, 1976; de Haan & Campell, 1991 | Duchaine, 2000 | Temple, 1992 | Nunn et al., 2001 | Kracke, 1994 | Ariel & Sadeh, 1996 | Jones & Tranel, 2001 | de Gelder & Rouw, 2000 | Bentin et al., 1999 |
| Age at testing | | 12, 27 | 52 | > 60 | 37 | 19 | 8 | 5 | 42 | 36 |
| Familial Involvement | | mother | relatives | cousin? | | father | | | | father |
| Brain Scans | | n | | | n | | | n | n | small RTL |
| EEG | | right posterior | | | | | Bi-occipital | | | N170 abn |
| IQ | Verbal | 144 | | 136 | 132 | 110 | 142 | 140 | | 127 |
| | Performance | 100 | | 147 | 135 | 105 | 90 | 110 | | 113 |
| Neurological | Motor coordination | poor | poor | poor | | poor | | n | | n |
| Testing | Hearing | tonal memory | CAPD | tone deaf | | | | | | |
| Face | Age | | | n | n | abn | abn | | | n |
| Perception | Gender | abn | | n | n | | abn | abn | | n |
| | Expression | abn | | | n | abn | abn | n | | n |
| | Face matching (BFRT) | 39 | 43, slow | 43 | 46, slow | n, slow | poor | | 34 | 41 |
| | Face memory (RMT Faces) | 28 | 46 | 43 | 41 | 24 | n | | 34 | 32 |
| | Covert Recognition | absent | | | | | | absent | | absent |
| Other visual | Basic visual processing | n | n | n | n | n | n | abn | n | n |
| abilities | Visuo-spatial abilities | abn | n | n | n | n | abn | abn | n | n |
| | Visual memory | n | n | abn | n | n | n | abn | n | n |
| | Object recognition | abn | n | n | n | | abn | n | n | n |

n = normal; abn = abnormal; RTL = right temporal lobe; CAPD = central auditory processing deficit.

## 8. Age and gender of subjects

Age of described cases at the time of testing varies between 5 years (case TA) and the 60s in the case of Dr. S. Only 2 of the 9 published cases are females. Whether this difference is meaningful, is an open issue.

## 9. Neurological Assessment

Not all case reports include data from comprehensive neurological examination. Five of the 9 reports provide information about brain scans, all of which are without clinically significant findings. In one study (YT [6]) volumetric MRI analysis was carried out. YT was found to have a smaller right temporal lobe, compared to control subjects.

Taken together, there is no clear evidence of overt structural abnormalities in DP. However, there might be subtle alterations which are not detectable by visual inspection of structural scans alone, and volumetric analysis methods may provide very useful additional information of brain areas affected in DP.

Abnormal EEG was reported in 3 patients. AB was found to show "moderate abnormality, including small spikes over the posterior part of the right hemisphere" [53]; LG showed abnormal findings on both occipital lobes [1]. Only one case was studied using event-related potentials (ERP). In YT ERPs did not differentiate between faces and other complex visual objects, such a difference being typical for healthy control subjects (e.g. [5]). It appears thus promising to administer ERPs to persons suffering from DP to gain insight into the underlying functional brain deficit.

There are a number of further subtle neurological abnormalities reported in DP. In the initial case report, Patient AB was described to have poor motor co-ordination and severely impaired motor proficiency. Patient BC [23] showed motor difficulties "most clearly manifest in a gait abnormality, and as a boy, he never enjoyed playing sports". In addition, he suffered from a central auditory processing deficit, which made it difficult for him to understand speech in noisy settings.

Similarly, patient HD reported by Kracke [48] showed substantial delays in motor development and "hated physical games at school". His gait at age 19 appeared to be ungainly, his handwriting immature and irregular. Dexterity tests revealed scores of one standard deviation below average and "his practical abilities are rated weaker than his theoretical knowledge".

Dr. S, described by Temple [68], complained of clumsiness and was reported to be tone deaf. Interestingly, AB was found to score poorly on a tonal memory test. McConachie cites a study by Milner [55] which yielded evidence for tonal memory deficits in a group of patients that underwent temporal lobectomy. AB's score was below the group mean of the lobectomy patients [55].

In patients YT and TA [45], a neurological examination did not yield any abnormalities. In cases VA [15], EP [58] and LG [1], neurological status was not assessed.

In summary, of the 9 cases, 3 were found to have difficulties in auditory processing (AB, BC, Dr S). The same 3 patients also showed motor deficiencies (gait abnormality, clumsiness). Taken together those neurological findings imply that brain scanning cannot substitute for a thorough clinical neurological examination in DP patients. In addition, assessment of auditory functions should be included.

## 10. General intellectual abilities

Of the 9 cases reported here, 7 were found to possess superior intellect (no IQ data for VA). The only case with an IQ below 120 was diagnosed as suffering from Asperger Syndrome; in this case an IQ of 108 seems still remarkable.

This pattern may be influenced by the fact that very intelligent people are more likely to seek medical or psychological assistance. It is still remarkable that all but one patient showed extraordinary intellectual abilities. It is also noteworthy that out of 7 patients for whom verbal and performance IQ were reported separately, 5 showed a higher verbal IQ, with a verbal/performance difference of more than 30 in 3 cases (52 in patient LG). A separate report of verbal and performance abilities may thus be of interest.

## 11. Basic visual abilities

A wide range of tests has been applied to analyse basic visual functioning in DP. Where visual acuity was tested formally, it was found to be intact (BC, EP, TA, YT). Colour vision tested formally in 5 cases (not in BC, Dr. S, HD, VA) did not yield any deficits. In 7 cases (all except BC and HD), perception of orientation was tested using variants of the Benton line orientation test [8]. Performance was normal or above average with one exception (borderline result in TA). Figure-ground segmentation was assessed in 4 patients (AB, BC, EP, YT) and found to be intact in all cases.

Basic visual abilities were unimpaired in all cases except TA, but should be screened comprehensively in DP cases.

## 12. Visuo-spatial abilities

Assessment of visuo-spatial abilities involves a wide range of procedures, and the available evidence is based on very different tests which restricts comparability across cases.

Topographical orientation problems in everyday life were reported in two cases (AB, LG). The Hooper visual organisation test was administered in 3 cases (LG, TA, YT) and yielded very poor results in cases LG and TA, and normal performance in case YT. When maps of familiar environments had to be drawn, AB scored poorly, LG was found to be accurate but very slow, while EP had no difficulties. Dr. S and LG showed normal mental rotation performance. The ability to draw common objects from memory was tested in patients AB, BC, EP, LG and TA and was impaired in TA only. AB's drawings were accurate but untidy and she reported "having great difficulties at school with drawing maps and mathematical figures and with copying biological diagrams". In TA, simple drawings (house, clock, alligator) were inadequate. He further showed deficits in the visual motor integration test, which requires copying geometrical objects, a task that was done perfectly by EP.

It appears that only the young patients had difficulties with visuo-spatial tasks. If tested, adults were found to show intact visuo-spatial abilities. This suggests that DP patients may learn to compensate for visuo-spatial deficits and that practice may lead to significant improvements.

## 13. Visual memory

Visual memory was mainly assessed using the Benton visual retention test. Impairments of AB were initially reported [53] but not confirmed later [17]. HD, YT, LG and VA scored in the normal or above-average range. EP showed normal memory for the Rey-Osterrieth figure.

TA's score on the Benton Test was borderline, while a wide range of memory and language functions were normal. The poor Benton score might reflect his visual deficits rather than memory impairment per se.

In Dr. S, a strong dissociation between verbal and visual memory emerged using the Wechsler Memory Scale (WMS [71]). While her verbal memory was superior or average, her visual memory was significantly impaired both in immediate and delayed design recall. Recall of the Rey Figure was also poorer than expected. Her visual memory scores are in striking contrast to her otherwise superior level of functioning. In BC visual memory was not formally tested. Taken together, evidence for an impairment of visual memory is only seen in Dr. S. Short screening of visual memory function in cases of DP is useful and should be included in assessment.

## 14. Object recognition

Object recognition skills were frequently assessed using the Boston Naming Test (Dr. S, EP, VA, YT; all normal results), the Birmingham Object Recognition Battery (BC, EP, VA, all normal results) and naming the Snodgrass and Vanderwart picture set (BC, VA; normal results).

In the follow-up study, AB was comprehensively assessed on a range of newly developed object recognition tasks. She was impaired in naming flowers and motorcars and in forced choice decisions about objects, e.g. concerning their familiarity. Moreover, she made significantly more errors in the Oldfield Wingfield test than control subjects. De Haan et al. [17] concluded that "AB shows a mild but generalised problem with visual object recognition and naming".

LG was tested with real objects and he was unable to recognize 10 out of 45 by vision alone. He recognized them, however, when he was allowed to touch them. He failed to identify common objects from coloured photographs in 43% of cases. With unusual views of objects, his error rate increased to 70%. When line drawings of objects were presented, he made only 10% errors, but his performance deteriorated when line drawings of objects overlapped. As with photographs, when objects were drawn from an unusual view, his performance was very poor (15% correct). Line drawings of objects that were partly covered produced an error rate of 72%. Although data of age-matched controls are not provided, the scores indicate a severe deficit in object recognition, in particular with ambiguous stimuli.

TA's ability to name visually presented real objects was reported to be above average. TA was also presented with 300 items drawn from the categories of animals, fruit/vegetables, tools/utensils, musical instruments and vehicles. His ability to name and identify the items was fully intact. Jones and Tranel [45] concluded that recognition and naming of non-unique entities was entirely normal in TA.

In addition to the Boston Naming Test, YT completed a wide range of additional object recognition tasks; his performance was normal or above average for real objects, overlapping line drawings, pictures of objects photographed from prototypic and unusual angles, partially masked, fragmented and incomplete drawings. Bentin et al. [6] concluded, "YT did not show any sign of a general agnosic problem in the visual modality".

In HD, object recognition was not formally tested.

In summary, of the 9 DP patients only 2 showed problems concerning object recognition, ranging from mild in AB to severe in LG.

In order to exclude a more general visual processing deficit, comprehensive assessment of object recognition is necessary, either by standard paper-and-pencil techniques or by computer aided testing of within-category discrimination including simultaneous response times measuring. Response time measurement is of potential value because it allows detection of speed-accuracy trade-offs that could result from prosopagnosic subjects feeling pressured to respond faster to faces (for a detailed discussion see [31]).

## 15. Face Processing

According to the Bruce and Young model [9], the first stage in face processing is structural encoding. During this process, a pictorial code that is generated for every visual scene is transferred into a more abstract representation ("structural code"). Simple face matching tasks can be performed on the basis of a pictorial code alone, using feature comparison strategies. Assessment of a subject's ability to structurally encode facial images should therefore involve matching two different photographs of the same face rather than two copies of the same photograph of a person.

Structural codes differ depending on the familiarity of a person. While familiar faces produce an "identityspecific semantic code" which is based on the person's own experience with a given stimulus, the code generated for unfamiliar faces is termed "visually derived semantic code" and has to rely on the physical properties of the face [9]. This facial representation can be used for any form of processing that can be carried out on unfamiliar faces, for example gender and age judgements, or perception of the emotional state of a face. An identity-specific semantic code can only be tapped if a presented face has been seen before. Activation of such stored experiences induces a "feeling of knowing" [9]. Prosopagnosia could thus be due to a failure in transferring a pictorial code into a structural code for further processing or due to intact face recognition units that do not activate person identity nodes. In recent years research has focused on a deficit in structural encoding of faces as the critical feature in prosopagnosia (e.g. [6,27]). This notion is based on the fact that many prosopagnosics are not only impaired in the recognition of famous faces, but also in processing stages relying on physical face information alone, like age, gender and emotional state discrimination.

Extensive assessment of face recognition abilities was completed in all case reports. However, studies differ substantially in the range of face processing domains that have been examined.

## 16. Face matching

The Benton and Van Allen Test of face matching (BFRT [7]) was administered to all patients except TA. Patients LG and VA showed a substantial deficit, while AB achieved a borderline score. BC, Dr S, YT and EP scored within the normal range, but BC and EP took a very long time (up to one minute per item). They seemed to be meticulously comparing every single feature of the stimuli. The same pattern was observed for HD on a matching task comparable to the BFRT.

For patients AB and BC, additional face matching tasks were designed. These tasks were more difficult than the Benton and van Allen Test, and yielded impaired performance in both patients relative to a control group.

The findings suggest that some DP patients are able to complete relatively easy tests of face matching, which do not require an intact face processing system. Increasing task demands and response time analysis, however, reveal face processing impairments. As reported elsewhere (see for example [24]), face matching can be achieved by comparing single features of faces or even by non-facial pictorial cues when identical stimuli are used. If face matching is tested, procedures with higher task demands and response time measurements are useful to detect changes in processing strategies.

## 17. Face memory

The Recognition memory test (RMT [70]) was administered to all patients except TA. In this test, a list of words and a list of faces are presented. After presentation of each list, pairs of words and faces are shown and subjects have to indicate the target for each pair.

Word recognition was above average in the 6 patients who completed both parts (poorest score was 47/50). In the face part, AB, HD, VA and YT were significantly impaired, BC, Dr. S and EP showed normal performance. LG was reported to show good performance on a comparable test. It should be noted that the RMT uses identical photographs on the study and the recognition lists. The clothes of the persons are visible in part and the items differ in background. The RMT is thus not a pure face memory test, and recognition is aided by non-facial cues.

## 18. Other face processing tests

Four patients (AB, Dr. S, EP and TA) were tested with the Mooney faces. Subjects have to look at patterns of light and shadow in black and white that represent faces. Their task is to judge the gender and age of the depicted person. The test requires the integration of black and white blobs into a coherent percept. While Dr. S and TA performed within the normal range, AB and EP were impaired relative to normal controls. Given the fact that both patients who performed poor in this test also revealed deficits in other tests of visual processing, it can be stated that the Mooney faces do not provide additional evidence.

A test that required naming of individual face parts indicated normal scores in EP, while LG did not achieve a single correct score.

An interesting task was recently introduced by Duchaine [23], the "one in ten task". Subjects are required to identify 15 photographs of the same person differing in illumination and angle among 150 photographs presented in succession. BC was significantly impaired in this ecologically valid task.

## 19. Identification of gender and age

Gender discrimination was tested using specially designed procedures in AB, EP, LG and TA. Only EP showed normal performance, while AB, LG and TA had great difficulties. Age discrimination was found to be normal in EP and very poor in LG, the other patients were not examined. YT was reported to be unimpaired at processing gender and age, although no formal testing was undertaken.

## 20. Emotional Expressions

Recognition of facial emotion was tested in 5 patients. EP's ability to name facial affect was found to be normal, and TA was able to match different faces showing the same emotional expression as well as control subjects. AB was slightly impaired at matching and more severely impaired in naming facial affect. HD and LG were markedly impaired in affect naming tasks, while YT had no problems.

## 21. Recognition of familiar faces

Identification of familiar faces was tested using two different procedures. In the younger patients (HD, LG, TA and AB in the initial testing), photographs of relatives and close friends of the families were used as stimuli. This procedure ensures that the people depicted in the photographs are really familiar to the subject. Shortcomings are the limited amount of available test items and the familiarity of the subjects with the photographs in addition to the faces. AB showed normal accuracy, but very poor response speed when assessed at age 12. HD identified all of his relatives in the photographs, but his performance deteriorated dramatically, when hairstyle was not visible in the stimuli. LG and TA were found to be very poor at recognizing relatives.

When photographs of famous people, like politicians, actors etc. were presented, all patients showed poor performance ranging form 3% in YT to 59% correct scores in AB. Tests of famous faces were all specifically developed for testing and differed substantially in difficulty and number of items. YT, for example, was tested with 670 famous faces, of which he recognized 3%. Control subjects recognized 58%. In tests with fewer items, performance of control subjects is usually above 90% because the average familiarity tends to be higher.

## 22. Covert face recognition

A remarkable aspect of face processing is the ability to implicitly recognize faces, without any conscious experience of recollection. Patients suffering from acquired prosopagnosia have been reported to show intact implicit markers of recognition, such as autonomic skin responses [4], event related potentials [60] or response time benefits [18].

Covert face recognition was examined in 3 of the DP patients. AB was tested with pairs of faces and names presented consecutively. The second face or name could be related, unrelated or neutral to the first (i.e. an unknown face). She exhibited a priming effect for names but not for faces. This indicates impaired covert face recognition.

Bentin et al. [6] presented faces together with names. The name could be either the name of the person whose face was shown, or a different person. The subject had to indicate whether the name belonged to a politician or a media celebrity. YT's reaction times did not differ between conditions, while control subjects were faster and more accurate in the matching condition. There was thus no evidence for implicit recognition in YT.

In TA, skin conductance responses (SCR) were registered while he was presented with a series of familiar (family and close friends) and unfamiliar faces. Although TA was unable to name any of the 13 familiar faces, his skin conductance responses to these stimuli were reliably enhanced, similar to the pattern seen in control subjects. Current models of covert face recognition (see [66]) assume that overt and behavioural covert recognition of faces make use of the same underlying system, while an arousal response to familiar faces might be mediated by a dorsal visual-limbic pathway as proposed by Bauer [4]. This idea receives confirmation by the pattern of results found for the patients suffering from DP. Behavioural measures do not suggest covert recognition in DP, while there is some evidence of covert recognition if autonomic responses are assessed.

## 23. Familial involvement

De Haan [16] presented the case of a family in which 2 daughters and the father showed severe impairments in a face recognition task, while a son and the mother showed normal results. This finding as well as occasional anecdotal reports of DP patients suggest a potential genetic basis of face processing deficits.

Five of the 9 DP cases reported that they have family members who have some difficulties in recognizing others by their faces. AB, BC, Dr. S, HD and YT are known to have close relatives who complain about face recognition problems. These observations strengthen the hypothesis of an involvement of genetic factors in developmental prosopagnosia, although there is as yet no direct evidence.

## 24. Discussion

In all cases described here, face recognition problems are disproportionately more severe than object recognition problems. Therefore all patients can be considered as fulfilling the criteria for prosopagnosia. However, in 4 out of the 9 patients, formal testing revealed evidence for deficits in non-facial visual processing. While in two patients (AB, LG) object recognition was poor, one patient (Dr. S.) showed deficits in visual memory, and patient TA showed deficits in basic visual processing. It is noteworthy that the 4 patients found to have difficulties with visual processing do not show a deficit in more than one processing domain (e.g. object recognition and visual memory). The additional impairments are thus quite specific rather than affecting all aspects of visual processing.

Severe impairments in recognizing other people on the basis of facial information are the core deficit in all patients. However, they differ substantially on other face processing functions. Face matching was found to be deficient only in 2 patients (LG and VA). When task difficulty was increased, 2 further patients were impaired. This finding suggests that simple tests of face matching are not necessarily sensitive to the detection of impairments in face processing (see also [24]). The same applies to tests of face memory, e.g. the face subtest of the RMT. Out of 8 patients, 4 achieved normal results. As these patients scored poorly on other face recognition tasks, it is conceivable that their RMT performance is not attributable to intact face recognition memory, they may rather use other features of the stimuli like background lightning, kind of dress or hairstyle to identify the targets.

## 25. Functional Subtypes of developmental prosopagnosia

An important question concerns the issue of variants or subtypes of developmental prosopagnosia, and their relation to processing stages. For acquired prosopagnosia, a distinction between apperceptive prosopagnosia and associative prosopagnosia has been suggested [20]. Patients suffering from apperceptive prosopagnosia have been found to show poor performance in perceptual face processing tasks (face matching, age estimation, etc.), while patients suffering from associative prosopagnosia are selectively impaired on tests requiring access to information about faces stored in memory (recognition of familiar faces).

The Bruce and Young model of face processing [9] assumes that processing of more basic face information takes a different route than face identification. While identification requires successful structural encoding, more basic processing like age and gender discrimination or analysis of facial affect can be carried out on the basis of view-centred descriptions that do not require a structural description. Of the cases discussed here, patients who had problems in judging gender and age of faces also tended to show deficits in extracting information on the affect of faces. In 3 out of 5 cases, this pattern was observed (AB, LG, HD). Patients who are impaired in basic face processing tasks are also impaired in other visual functions. AB and LG had difficulties with object recognition and TA was impaired in basic visual functions.

This pattern suggests a classification of DP patients into two categories. The first category comprises patients who are impaired in basic face processing and also show deficits on other forms of visual processing (AB, LG, TA). The second category represents cases with pure face recognition impairments in the presence of intact basic visual processing. Note that the classification suggested here does not map onto the classification criteria suggested for acquired prosopagnosia. The distinction between apperceptive and associative prosopagnosia is based on performance on tests of unfamiliar face matching and directed visual processing [20]. We suggest that patients who are unable to carry out tasks of directed visual processing with faces (age estimation, judgement of facial affect) fail to obtain view-centred descriptions, which are, according to the Bruce and Young model [9] precursors of the more abstract expression-independent descriptions, which are the product of structural encoding. View-centred descriptions do not seem to be specific for faces, as the patients with impairments of processing the physical aspects of faces also show difficulties in non-facial tasks like object recognition or tests of visuo-spatial abilities. In tests of face matching, only 2 of the cases fall below the cut-off score (LG, VA). However, as difficulty is increased, 2 further patients show deficits (AB, BC). Moreover, 3 further cases process the task very slowly, comparing individual features separately, or have borderline scores (EP, TA, YT). Apart from Dr. S., all cases appear to show face matching deficits. Patients who score poorly also have problems with object recognition or more basic visual deficits, and their poor performance might be attributed to the missing compensation of non-specific processing mechanisms that are able to contribute to face matching. Three cases have difficulties in carrying out simple face processing tasks (AB, LG and TA) while 5 have difficulties with perceptual processing. Only Dr. S. seems to be impaired at the level of recognition and therefore fulfils the criteria for associative prosopagnosia. Developmental Prosopagnosia is not a unitary syndrome; and there is as yet only limited evidence for a classification into different subtypes.

## 26. Face specificity

Whether prosopagnosia represents a disorder that is specific to faces is a matter currently under debate [11,46,62,67]. Although face processing has been considered as a specific module for a long time, recent evidence suggests that face and object recognition may make use of the same underlying mechanisms.

The evidence from the DP cases reviewed here does not clearly support one or the other hypothesis. Of 9 patients, 5 (BC, EP, HD, VA, YT) seem to show deficits solely in face processing tasks. In HD and VA object recognition was not comprehensively assessed, therefore these cases only produce limited support for face specific deficits. In 4 cases (AB, Dr S., LG, TA), visual processing deficits were not confined to face stimuli. AB was found to be impaired in within-category discrimination, which has been discussed as the underlying deficit in prosopagnosic patients [31]. Dr S. had problems with visual memory, and LG and TA both had problems with a range of visual processing tasks. It should be noted that both were below ten years of age (5 and 8 respectively) and their perceptual abilities might change during development due to maturation and training. In the case of AB, a poor score on the Benton Visual Retention Test in an initial report, was followed by a normal score 15 years later.

A future follow-up study of LG and TA would possibly yield interesting insights into changes of perceptual skills over the lifetime of prosopagnosic patients. There is little evidence in favour of plasticity in face recognition. Recent evidence [50] suggests that experience with faces has to occur within a critical period. Infants born with a dense central cataract in both eyes leading to reduced visual input until corrective surgery was carried out between the second and sixth month of age showed significant deficits in face processing tested nine or more years after surgery. Thus, experience with face stimuli during the first few months of life appears to be critical for the development of normal face processing skills.

## 27. Autistic traits in DP?

Important information on the biological basis of DP can also be derived from the study of patients with autistic spectrum disorder (ASD). Deficits in face recognition seem to be more common in people suffering from autism or Asperger syndrome than in any other clinical population. Alterations in brain activity have been documented while high-functioning individuals with autism or Asperger syndrome discriminated between different faces and objects [65]. While there was reduced activity in the fusiform gyrus, more activation was observed in the inferior temporal gyrus yielding a pattern "that is consistent with feature-based strategies that are more typical of non-face object perception" [65]. It has been suggested [35] that specialisation of brain regions involved in face recognition is deficient because of lack of interest in social interaction and therefore the developing face recognition system is provided with an insufficient amount of input. Three of the published cases indicate autistic symptoms, while two reports do not comment on this issue and three state that there are no such symptoms. The fact that face recognition deficits are closely related to autism is well documented (see [35]). Two different explanations can account for the relation of DP and ASD. First, cases of DP could resemble a very mild expression of an autistic disorder that manifests itself solely in face recognition deficits. Second, subjects with autistic symptoms form a subgroup among cases of DP reported here. There is as yet inconclusive evidence for either of these hypotheses.
